# Development and validation of an artificial intelligence-powered acne grading system incorporating lesion identification

**DOI:** 10.3389/fmed.2023.1255704

**Published:** 2023-10-06

**Authors:** Jiaqi Li, Dan Du, Jianwei Zhang, Wenjie Liu, Junyou Wang, Xin Wei, Li Xue, Xiaoxue Li, Ping Diao, Lei Zhang, Xian Jiang

**Affiliations:** ^1^Department of Dermatology, West China Hospital, Sichuan University, Chengdu, China; ^2^Laboratory of Dermatology, Frontiers Science Center for Disease-related Molecular Network, West China Hospital, Clinical Institute of Inflammation and Immunology, Sichuan University, Chengdu, China; ^3^Med-X Center for Informatics, Sichuan University, Chengdu, China; ^4^College of Computer Science, Sichuan University, Chengdu, Sichuan, China

**Keywords:** dermatology, acne, artificial intelligence, acne lesions, grading system

## Abstract

**Background:**

The management of acne requires the consideration of its severity; however, a universally adopted evaluation system for clinical practice is lacking. Artificial intelligence (AI) evaluation systems hold the promise of enhancing the efficiency and reproducibility of assessments. Artificial intelligence (AI) evaluation systems offer the potential to enhance the efficiency and reproducibility of assessments in this domain. While the identification of skin lesions represents a crucial component of acne evaluation, existing AI systems often overlook lesion identification or fail to integrate it with severity assessment. This study aimed to develop an AI-powered acne grading system and compare its performance with physician image-based scoring.

**Methods:**

A total of 1,501 acne patients were included in the study, and standardized pictures were obtained using the VISIA system. The initial evaluation involved 40 stratified sampled frontal photos assessed by seven dermatologists. Subsequently, the three doctors with the highest inter-rater agreement annotated the remaining 1,461 images, which served as the dataset for the development of the AI system. The dataset was randomly divided into two groups: 276 images were allocated for training the acne lesion identification platform, and 1,185 images were used to assess the severity of acne.

**Results:**

The average precision of our model for skin lesion identification was 0.507 and the average recall was 0.775. The AI severity grading system achieved good agreement with the true label (linear weighted kappa = 0.652). After integrating the lesion identification results into the severity assessment with fixed weights and learnable weights, the kappa rose to 0.737 and 0.696, respectively, and the entire evaluation on a Linux workstation with a Tesla K40m GPU took less than 0.1s per picture.

**Conclusion:**

This study developed a system that detects various types of acne lesions and correlates them well with acne severity grading, and the good accuracy and efficiency make this approach potentially an effective clinical decision support tool.

## Introduction

Acne vulgaris is the eighth most prevalent disease affecting 9.4% of the global population (1). Although acne can occur at all ages, adolescents are the most prevalent group of acne sufferers, and eighty-five percent of adolescents are affected by acne (2). As a condition that alters appearance, acne affects patients’ physical and psychological well-being and causes a strong desire for treatment (3). The large patient population and the strong desire for treatment seriously burden healthcare resources (4, 5). Assessment of acne severity is essential for the patient’s stepwise therapy. There are more than 20 published scales for evaluating acne, but none is adopted universally for clinical practice (6).

Most scales can be classified as lesion-counting scales or text description scales. Lesion counting scales correspond to the severity by measuring different types of acne lesions, such as the Global Acne Grading System (7, 8). Counting acne lesions is supposed to be a more objective method. However, it shows a high degree of variability between raters due to ambiguity between different categories of skin lesions and interevaluator differences in the definition of skin lesions (9). In addition, a single counting process ignores the degree of inflammation, postinflammatory hyperpigmentation, scarring, and other features that affect the severity. In contrast to quantitative scales, qualitative scales distinguish between different levels of severity through textual descriptions. Although qualitative scales require more clinical experience from the evaluator, they simplify the tedious counting process to a certain extent and take care of other acne characteristics beyond the number of lesions. For example, Investigator Global Assessment classifies acne into five levels through text descriptions (clear, almost clear, mild, moderate, severe, and very severe) (10). On this basis, a recent study found that replacing the qualitative labels with the corresponding treatment intensity labels effectively reduced the high interrater variability, although these labels are more unstable since treatment options may change depending on regional perceptions and disciplinary developments (11).

Artificial intelligence (AI) for acne grading has been considered a promising research direction to increase the consistency and efficiency of assessment. Some AI systems focus on identifying and counting different types of lesions, but as with lesion-counting scales, they ignore considerable information beyond the countable lesions (12, 13). Other AI systems analyze the image as a whole but leave the evaluation free from clinical interpretability (14, 15). We believe that the quantity of different types of lesions is an inadequate but crucial component of acne severity assessment. Therefore we sought to develop a novel AI system that could integrate the identification and counting of skin lesions into the overall facial evaluation process, thereby improving the predictive accuracy.

## Materials and methods

### Database

This study was conducted at sichuan university from January, 2020 to June, 2022, and was approved by the west china hospital institutional review board to use the patients’ deidentified images and records. This study followed the declaration of Helsinki and standards for reporting of diagnostic accuracy (STARD) reporting guidelines and the checklist for evaluation of image-based artificial intelligence algorithm reports in dermatology (CLEAR Derm) (16). We collected records of 3,098 visits to our dermatology specialist clinics with a diagnosis of acne without other inflammatory skin disease diagnoses. Of the 3,098 visits recorded, 1,501 had corresponding standardized pictures obtained via the VISIA system, including frontal, left and right profile photos, and information from these visits was included in the current study. To select labeling experts for the database and to evaluate the adequacy of the standardized frontal photo, 40 patients with acne (10 mild, 20 severe, 10 severe) were selected based on clinical records. seven experienced dermatologists first rated the frontal photos of the 40 patients, and the three evaluators with the highest average linear weighted Cohen’s κ were selected to complete the severity marking of the 1,461 records. The median of their ratings was considered the true label. After disrupting the order of the 40 images, the 7 dermatologists again rated the combined photos (frontal and left and right side photos) of the 40 patients. To improve interrater agreement, in this study we used the Treatment Intensity label to distinguish between the severity of patients (11), and due to the low number of extremely severe cases, we combined Level 8 and Level 9 (Table 1).

**TABLE 1:** Severity label and corresponding treatment intensity list.

Grading label	Severity description	Treatment intensity
1	Clear	No treatment necessary
2	Almost clear	BPO or a mild topical retinoid
3	Mild	BPO and a topical retinoid
4	Mild to moderate	BPO and a stronger topical retinoid or a topical retinoid and consideration of an oral antibiotics
5	Moderate	Topical treatment and an oral antibiotics
6	Moderate to less severe	Same as 5, but start considering isotretinoin
7	Less severe	Same as 5, but recommend isotretinoin
8	Severe or very severe	Should be on isotretinoin

BPO, benzoyl peroxide.

### Development of the skin lesion identification platform

For the acne detection module, we used a publicly available deep-learning method to detect acne lesions (17). We used a VISIA complexion analysis system to photograph 276 facial images as our samples, where each sample has a resolution from 3128 × 4171 to 3456 × 5184 pixels. All the samples were split 9:1 into training samples (*n* = 248) and test samples (*n* = 28). Six dermatologists participated in annotating all the samples. A total of 15,922 skin lesions with 10 lesion categories, i.e., *open comedone*, *closed comedone*, *papule*, *pustule*, *nodule/cyst*, *atrophic scar*, *hypertrophic scar*, *melasma* and *nevus* were generated. Next, the network is trained by an SGD optimizer with 15 epochs, where the learning rate, momentum, and weight decay were 0.002, 0.9, and 0.0001, respectively.

### Development of acne grading systems

We used ResNet50 as the training network for the baseline results (18). This network contains four large blocks, each with 3, 4, 6, and 3 small blocks, and each small block consists of three convolutional layers. In addition, the network contains jump connections to alleviate the problem of gradient explosion and gradient disappearance during training, thereby allowing the model to extract deeper features. A total of 1,185 images were used for the grading experiments, of which, 945 were used for training and 240 for testing. For the training set, all images were first resized to 256 × 256 pixels and later randomly cropped to 224 × 224 pixels to meet the input size of the network. Furthermore, the images are randomly flipped horizontally (50% probability) and randomly rotated from −20° to +20° to expand the data to prevent training overfitting. The model was trained using cross entropy loss with a total of 200 epochs and a batch size of 32. The initial learning rate was 0.001, and it decayed to 0.0001 using a cosine annealing function. The optimizer was the Adam optimizer with a weight decay of 0.0001. The training was conducted on a Tesla K40m GPU. For the acne grading task, the number of acne lesions as well as the overall assessment are an important reference for acne grading. Therefore, we propose a method that combines dermatologists’ *a priori* knowledge with a CNN to automatically grade pictures. The acne counts of all samples were semiautomatically labeled by the trained detection model and manually validated by an experienced dermatologist. The rule divides each image into a grading interval instead of a single grade to guide the network to better predict the image grading.

**FIGURE 1:**
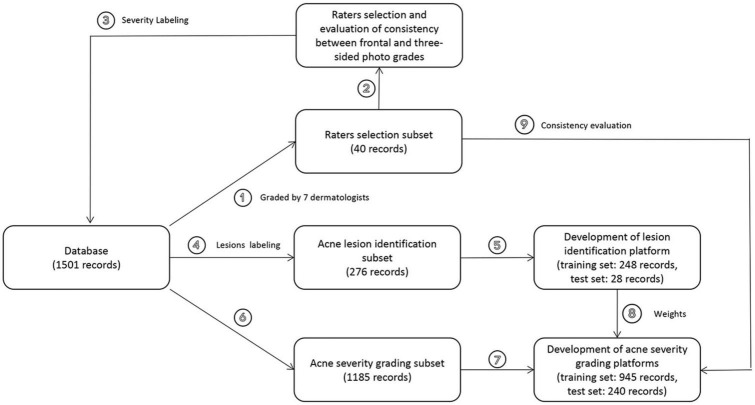
Overview of the development and validation of our AI systems. After confirming the adequacy of the frontal photo information, the doctors with the highest agreement with other peers were selected as true label raters for the remaining 1,461 frontal photos. Of the 1,461 photos, 276 were used to develop a skin lesion identification platform and 1,185 were used to develop an acne severity rating system. Then, we sought to incorporate skin lesion identification results into the severity evaluation and validated the feasibility in test set and rater selection subset.

We propose two methods to integrate the proposed rules into the network, i.e., fixed weights and learnable weights, and the two methods are shown in Figure 1. For the fixed-weights approach, the probability weight of the interval is fixed. If the interval does not contain the grading, the weight is 0; otherwise, it is 1. Each input image is fed into the CNN first to learn the image features. The image features are average-pooled and mapped to an 8-dimensional vector to correspond to the probability of each classification. Then, the two vectors are multiplied by the corresponding position elements to obtain the predicted probability of each classification. Since the proposed rule reduces the weight of the intervals that do not belong to the image classification, only the predicted probability of the interval to which the image belongs is obtained. The classification corresponding to the highest probability is selected as the predicted class.

**FIGURE 2:**
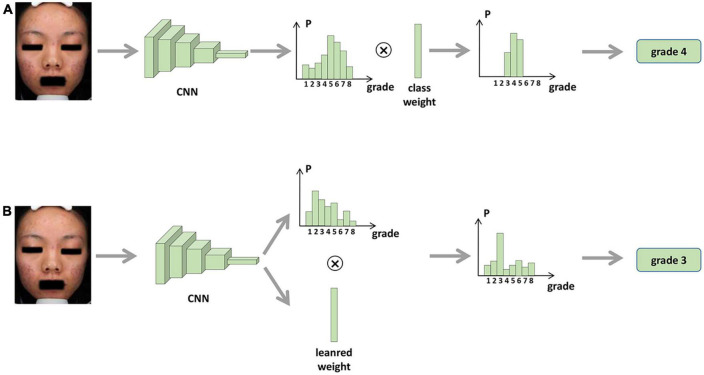
Procedure for integrating skin lesion identification with acne severity assessment based on AI. **(A)** Fixed weights approach. **(B)** Learnable weights approach.

For the learnable weights approach, the network is given an initial value, after which the weights are fine-tuned through training. As shown in Figure 2, after training, the network outputs the graded probability values and the learned interval weights. The prediction probability of each classification is obtained by multiplying the classification probabilities with the corresponding interval weights. Again, the classification with the highest probability is the grading predicted by the model.

### Statistical analysis

To determine the sample size of rater selection, assuming the interrater correlation coefficients were approximately 0.8, at least 7 raters and 40 subjects were needed. No formal sample size was calculated for validation of AI systems. Cohen’s kappa with linear weights was used to evaluate the AI’s performance against the true label or the 7 dermatologists on the rater selection dataset. A kappa value of less than 0.6 was considered unacceptably low. The statistical analyses were performed using Prism software (GraphPad Prism 8.0) and R (version 4.2.1).

## Results

The database was divided into three subsets, and the baseline characteristics are summarized in Table 1. Forty records were enrolled to select the true label rater. The mean age of the 23 female and 17 male patients was 24.8 years, ranging from 16 to 39 years. Of the 560 assessments (7 raters, 40 patients and 2 rounds), each grading of severity was represented by at least 2 subjects. The evaluations obtained through the frontal photos are in good agreement with those obtained through the three-sided photos, indicating that the frontal photos are sufficiently informative as samples for the AI evaluation (Table 2). For interrater agreement of frontal photo assessment, the pairwise Cohen’s kappa for each dermatologist ranked in descending order is shown in Supplementary Figure 1, and the three raters with the greatest average kappa value were selected to rate all the photos in the database. For consistency of the assessment of frontal photographs and 3-side photographs, the overall ICC for frontal photo assessment and 3-side photograph was 0.878 (0.814, 0.916), which suggests that a frontal photograph taken with VISIA alone can yield a similar amount of information for acne as three-sided photos.

**TABLE 2:** Baseline characteristics.

	Rater selection subset (*n* = 40)	Lesion identification subset (*n* = 276)		Severity grading subset (*n* = 1185)	
		Training subset (*n* = 248)	Test subset (*n* = 28)	Training subset (*n* = 945)	Test subset (*n* = 240)
**Age, years**					
<20	4	43	3	190	45
20–29	29	164	23	633	161
30–39	5	39	2	109	30
40–49	2	2	0	13	4
**Sex**					
Female	23	165	19	621	142
Male	17	83	9	324	98
Severity (true label)					
Clear	/	5	1	34	11
Almost clear	/	28	3	178	37
Mild	/	87	7	321	94
Mild to moderate	/	57	5	172	40
Moderate	/	41	3	149	35
Moderate to less severe		20	1	68	15
Severe	/	7	1	14	9
Severe or very severe	/	3	0	9	3

**FIGURE 3:**
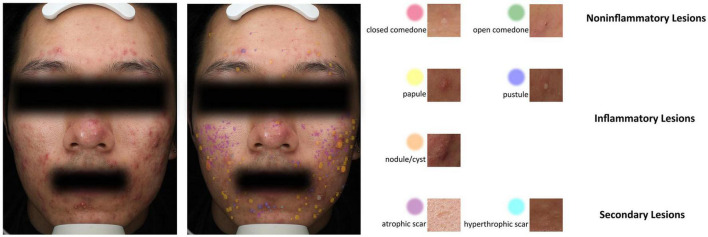
An example of seven types of acne-related lesions identified by the Lesion Recognition System in a patient with moderate to less severe acne.

For the development of the acne lesion identification platform, 276 frontal photos were labeled by five doctors and reviewed by a senior doctor. In total, 3,060 closed pimples, 2,192 open pimples, 3,861 papules, 884 pustules, 113 nodules or cysts, 5,410 atrophic scars and 302 hypertrophic scars were marked in 276 images (Figure 3). The 276 images were divided into a training set and a test set at a ratio of 9:1. The average precision of our model for skin lesion identification was 0.507, and the average recall was 0.775, which outperformed state-of-the-art one-stage and two-stage generic object detection methods. As previously anticipated, skin lesion counts are not sufficient for severity determination, and we were not able to build a decision tree model with good performance for acne severity evaluation, either based on the number of manually annotated lesions or the number of lesions identified by the algorithm (data not shown). However, different types of lesions have different distribution patterns on the face (Supplementary Figure 2). Inflammatory lesions (papules, pustules, nodules/cysts) are more evenly distributed, and non-inflammatory lesions and secondary lesions have unique distribution characteristics. Closed acne tends to be located on the forehead and midface, while open acne tends to cluster on the forehead. Atrophic scarring is concentrated on both cheeks, while hyperplastic scarring often occurs on the skin of the lower jaw.

**FIGURE 4:**
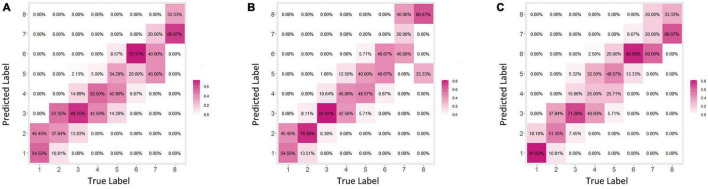
Confusion matrices for acne grading. **(A)** Original model. **(B)** Fixed weight model. **(C)** Learnable weight model.

**FIGURE 5:**
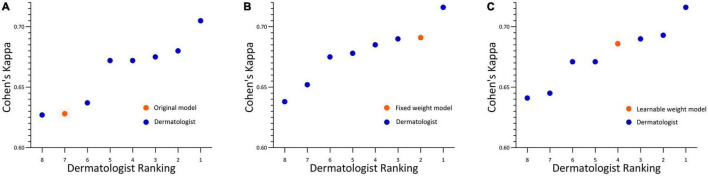
Acne grading performance on rater selection subsets. **(A)** Original model. **(B)** Fixed weight model. **(C)** Learnable weight model. Linear weighted Cohen’s kappa for each pathologist ranked from lowest to the highest. Each kappa value is the average pairwise kappa for each of the dermatologists compared with the others. The AI is highlighted with an orange dot.

For the development and validation of the severity grading systems, totally 945 images were used for training and 240 for testing, and the kappa obtained by the AI system relative to the true label was 0.652 (Figure 4A). To further enhance the predictive power, we further constructed a fixed-weight model a learnable-weight model to integrate the lesion identification results of papule, pustule and nodule/cyst into the severity assessment based on lesion identification platform, which improved the kappa relative to the true label to 0.737 and 0.696, respectively (Figures 4B, C). The 40 images that were initially used to select database annotators were applied to the three models, and the mean pairwise kappa achieved by the three AI models ranked 7th, 2nd and 4th (Figure 5).

## Discussion

In this study, we found that the artificial intelligence acne severity evaluation system we developed produced a reasonable evaluation of the frontal part of acne patients’ photos, and its evaluation results were in good agreement with the true labels. Furthermore, we innovatively incorporated the lesion identification results into the severity evaluation with fixed weights and learnable weights, which improved the performance of the model. The AI system, whether weighted or not, can grade acne within the performance range of experienced dermatologists.

Artificial intelligence has powerful learning capabilities that enable it to capture the nuances of lesion images, including size, color and texture, etc (19). The morphological manifestation of the lesion is an important basis for diagnosing and evaluating dermatologic diseases, making AI even more distinctive in dermatology (20). Currently, AI research in dermatology is focused on multiclassification tasks (21, 22) for disease diagnosis and binary classification (23, 24) for benign or malignant skin lesions, but the evaluation of the severity of a specific disease is also a research direction with great potential for application. The high prevalence and the lack of widely accepted evaluation criteria make acne a perfect fit for AI research. As the eighth most prevalent disease in the world, acne creates a medical need that cannot be met due to the current shortage and uneven distribution of dermatologists. AI can act as a decision aid for clinicians to improve the efficiency of evaluation, particularly in the identification and counting of acne lesions. In recent years, many advances have also been made in the evaluation of acne by AI. Sophie Seité made several optimizations to their model to improve the recognition of inflammatory and non-inflammatory acne lesions, and their model achieves a GEA score similar to that of the dermatologists (13). Quan Thanh Huynh applied different models to complete the identification of acne lesions and the evaluation of severity with good accuracy, but their study did not incorporate the results of lesion identification into the severity evaluation (12). To the best of our knowledge, no previous studies have integrated skin lesion identification with severity assessment and consequently improved the accuracy of severity assessment. According to the principles of AI, skin lesion identification may no longer be important for severity evaluation when the sample size is sufficiently large, however, for more limited sample sizes, lesion identification can emphasize important information in the evaluation of severity and make the results more interpretable by doctors.

One of the major strengths of our study is that we have a much more detailed classification of severity (eight scales) than what is used by other common scales. One study found that the interobserver agreement using a crude acne severity scale was quite low (25). In order to improve interrater agreement, we referenced the treatment intensity label used by the Elena Bernardis’s study to represent acne severity (11). The physicians in this study strongly endorsed the logic of this intensity label after discussion, although it differed slightly from the current Chinese Guidelines for the Management of Acne Vulgaris and medication habits of Chinese dermatologists. The use of treatment intensity for labeling, in addition to increasing interrater consistency, provides doctors with an indication of the patient’s treatment regimen. However, the doctors will need to take into account other information about the patient as well as the results prompted by the AI, because our model does not consider patient information outside of the image data, including but not limited to pregnancy and breastfeeding status, drug allergy history, financial situation, personal wishes etc. In addition we are more rigorous in testing of the models. Besides comparing the differences between the AI model predictions and the true labels, this study compared the AI predictions with the ratings of several experienced dermatologists. This step is important for grading systems that lack objective indicators such as acne severity.

Our study also suffered from a number of shortcomings. First, all of the patients we included were Chinese, and although there were different ethnic groups, all of the patients had skin types II to IV; thus, further validation of our model’s ability to identify lesions and evaluate severity in patients with other skin types is needed. Second, our samples were sourced from hospital specialist clinics, and due to the low willingness of mild patients to seek treatment and the small proportion of patients with extremely severe illnesses, our sample is not evenly distributed at different levels. Finally, to obtain more reliable results, we included only patients with a diagnosis of acne and no other facial inflammatory diseases; however, in the real world acne is not exclusive to diseases such as rosacea and seborrheic dermatitis, and the AI evaluation for this group of patients requires a broader sample resource.

## Conclusion

This study developed a system that detects various types of acne lesions and correlates them well with acne severity grading, and the good accuracy and efficiency make this approach potentially a very effective clinical decision support tool. However, further research is needed to validate the effectiveness of this AI system in real-world clinical settings.

## Data availability statement

The original contributions presented in this study are included in this article/Supplementary material, further inquiries can be directed to the corresponding author.

## Ethics statement

This study was approved by the West China Hospital Institutional Review Board to use the patients’ de-identified images and records.

## Author contributions

JL: Data curation, Formal analysis, Investigation, Methodology, Project administration, Resources, Validation, Visualization, Writing – original draft. DD: Conceptualization, Data curation, Formal analysis, Investigation, Methodology, Project administration, Validation, Writing – original draft. JZ: Methodology, Project administration, Software, Writing – original draft. WL: Methodology, Project administration, Software, Writing – original draft. JW: Methodology, Project administration, Software, Writing – original draft. XW: Methodology, Project administration, Software, Writing – original draft. LX: Conceptualization, Investigation, Writing – original draft. XL: Investigation, Writing – original draft. PD: Investigation, Writing – original draft. LZ: Software, Supervision, Writing – review and editing. XJ: Funding acquisition, Resources, Supervision, Writing – review and editing.
