# Supplementary material for: Development and validation of an artificial intelligence-powered acne grading system incorporating lesion identification
Source: Front Med (Lausanne). 2023 Oct 6;10:1255704. doi: 10.3389/fmed.2023.1255704 (PMC10587552; doi:10.3389/fmed.2023.1255704)
Supplement: Supplementary file 1 [file Data_Sheet_1.docx]

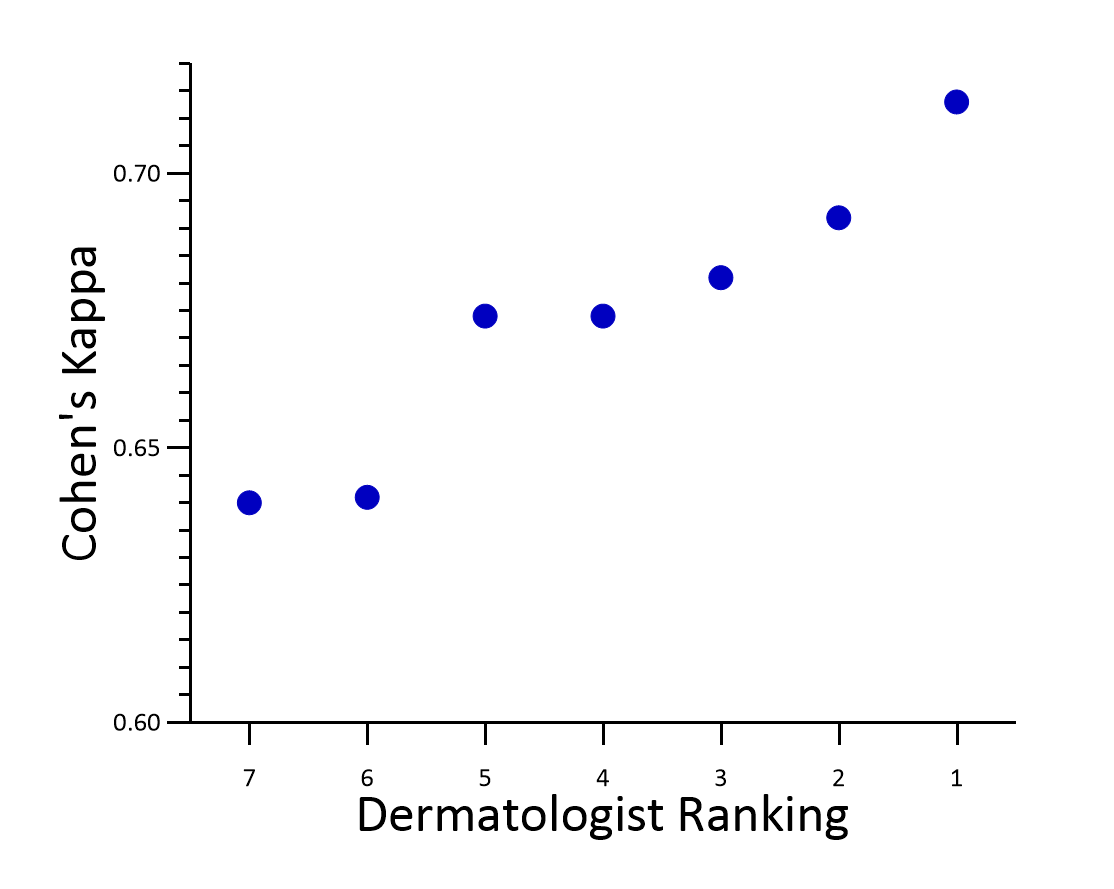


Supplementary Figure 1. Pairwise Cohen’s kappa for each dermatologist’s frontal photo assessment ranked in descending order.


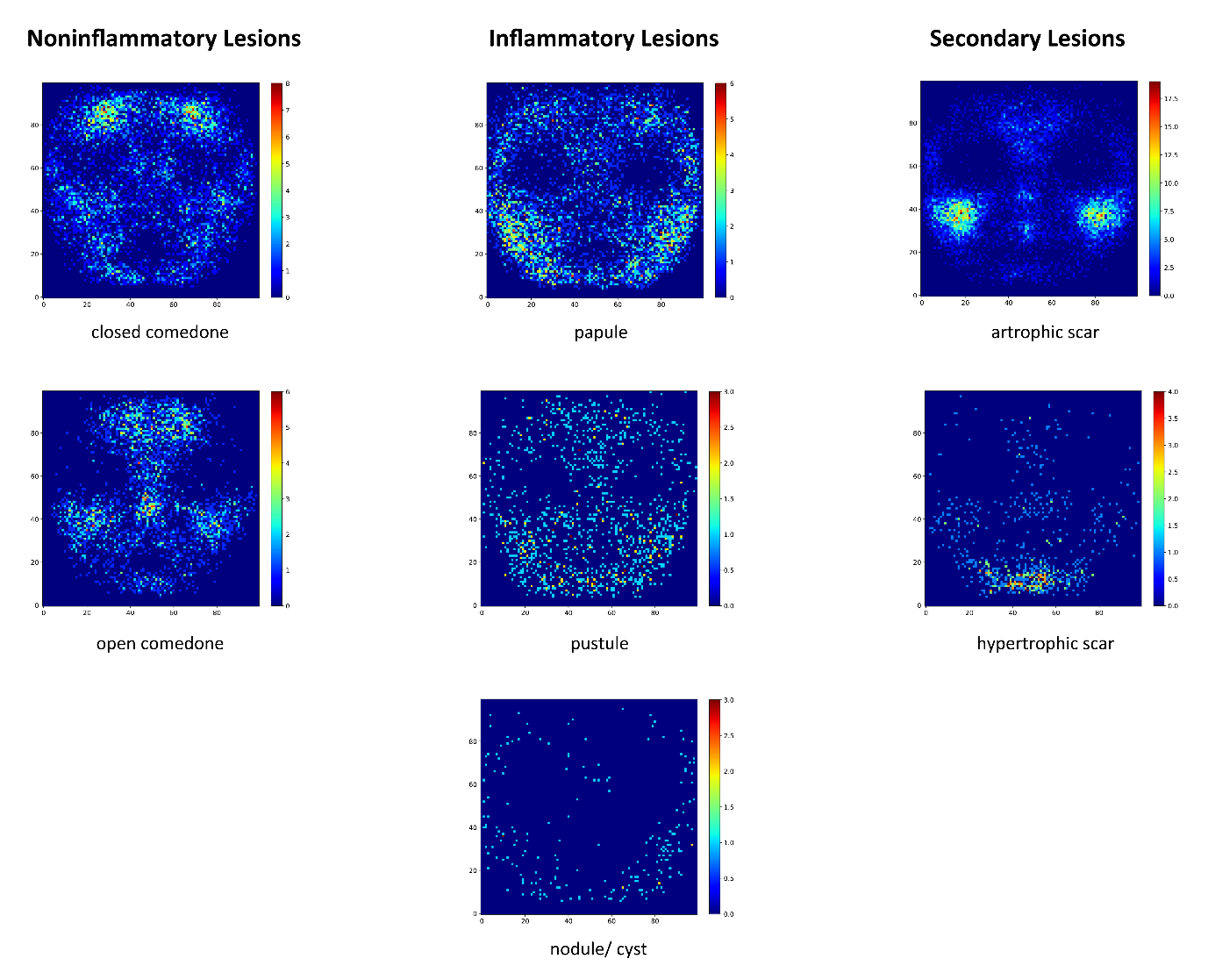


Supplementary Figure 2. Distribution patterns of acne-related lesions.
